# Edible coatings and lipid oxidation data in walnuts

**DOI:** 10.1016/j.dib.2021.107295

**Published:** 2021-08-18

**Authors:** Eduardo Caballero, Daniela Bernal, Jeniffer Wilckens

**Affiliations:** Centro Regional de Estudios en Alimentos Saludables-CREAS, Av Universidad 330, Valparaíso, Chile

**Keywords:** Walnuts, Edible coating, Acid value, Lipid oxidation

## Abstract

The information presented is part of an investigation that seeks a better understanding of lipid oxidation in walnuts. The data shown regarding edible coating, are one of the strategies used to investigate the effect over oxidation stability. For the present experiments, unshelled walnuts were coated with different formulations, and then stored at 37 °C, 20% RH for 6 weeks. After that time, coated nuts were taken out, cold pressed to extract the oil and analysed. The main data obtained from the oil analysis of walnuts were acid value, peroxide value, and thiobarbituric acid reactive substances (TBARS). Data show the variation of the parameters during the storage time at 37 °C, considering the different formulations of edible coatings and the control. These data are relevant to walnuts exporters to have a comparison point.

## Specifications Table

**Table:** 

Subject	Agricultural Sciences, Food Science: Food Technology.
Specific subject area	Edible coating technology as a protocol to measure parameters related with lipid oxidation in walnuts.
Type of data	Graph
How data were acquired	Spectrophotometer Jasco V-630. Manual Titration.
Data format	“Raw” “Analyzed”
Parameters for data collection	Unshelled walnuts (*Juglans regia L.*), Chandler variety located in lat. 34°16′43″S, long. 70°48′42″W, altitude 416 m, were cover with different edible coatings (including a plasticizer, a thickener, and an antioxidant), and left at 16 °C for 24 h before packaging. Some samples were packaged and another unpackaged. All samples were incubated at 37 °C (20% Humidity) during 6 weeks. Samples were analysed before incubation at 37 °C and after 6 weeks.
Description of data collection	Walnuts with different edible coatings and without edible coating (control), were analysed before incubation and after 6 weeks of incubation at 37 °C. Samples were cold pressed to extract the walnut oil. Walnut oil was used to measure acid value, peroxide value and thiobarbituric acid reactive substances (TBARS). Each experiment condition was carried out in triplicate. “Secondary Data”.
Data source location	Institution: Centro Regional de Estudios en Alimentos Saludables-CREAS City/Town/Region: Valparaíso Country: Chile Latitude and longitude (and GPS coordinates, if possible) for collected samples/data: lat. 34°16′ 43″ S, long. 70°48′ 42″ W, altitude 416 m. Raw data are in repository: Mendeley Data.
Data accessibility	Repository name: Mendeley Data Caballero-Valdés, Eduardo (2021), “Edible coating of walnuts. Final results”, Mendeley Data, V1, doi: 10.17632/rsvsbvnvr4.1 Direct URL to data: http://dx.doi.org/10.17632/rsvsbvnvr4.1

## Value of the Data

• Data related are important because shows the relation between edible coating and packaging over lipid oxidation (peroxide value and TBAR) or lipid hydrolysis (acid value) in walnut.
• Walnut's exporters, specifically companies related with Chilenuts, can be benefited from these data because they can focus their efforts in other developments to improve or maintain the walnut quality.
• This data can be used to reformulate edible coatings and packaging to explain the lipolysis and oxidation mechanisms in walnuts.

## 1 Data Description

**Fig. 1:**
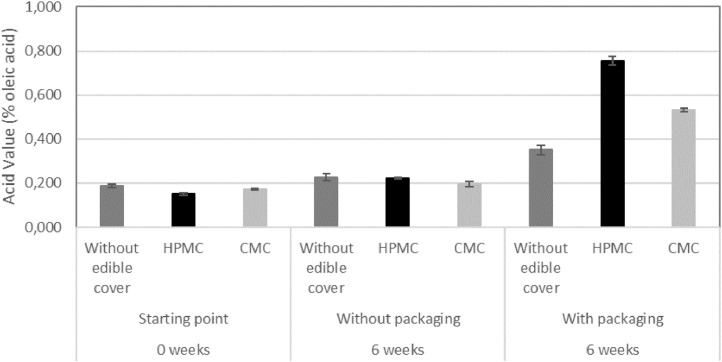
Acidity value, shown as free oleic acid percentage in walnut samples. *N*=3.

One of the walnut quality parameters considered for the present study was the acidity value, expressed as free oleic acid percentage (OA%). At the beginning of the experiment, coated and uncoated walnuts showed an acidity value of 0.16%. After the six weeks’ incubation time, all samples which remained unpackaged for this period, showed no significant change in this parameter, the value remaining at around 0.2%; while in the packaged samples, there could be seen a more significant variation. Samples packaged and uncoated showed a slight rise to 0.32%, HPMC-coated walnuts showed a rise to 0.8% of free oleic acid and CMC-coated walnuts presented a 0.49% free oleic acid when analysed. Considering this data, Fig. 1 is presented.

**Fig. 2:**
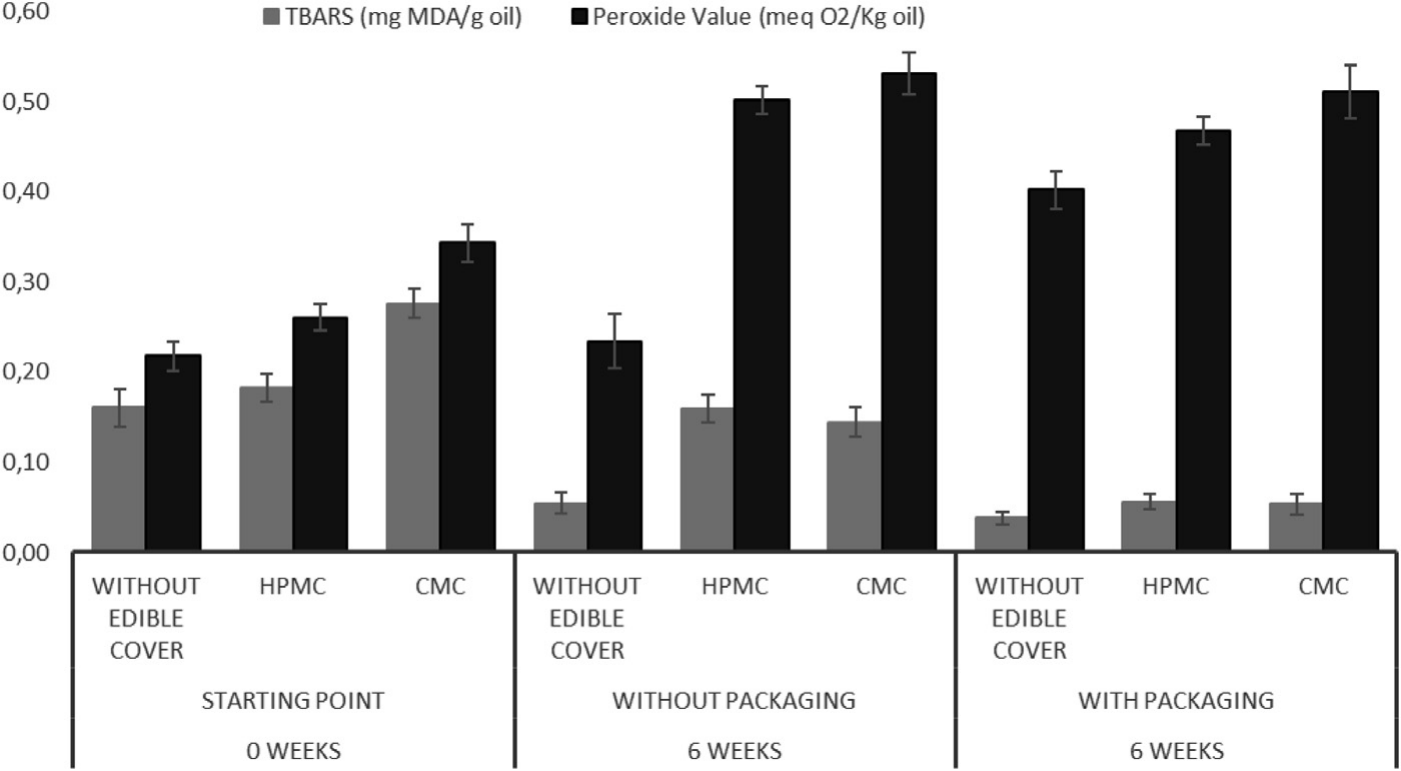
Primary and secondary oxidation values for walnut samples. *N*=3.

Other parameters to define the lipid oxidation stability are peroxide values as primary oxidation indicator and thiobarbituric acid reactive substances (TBARS) as a secondary oxidation indicator, shown as meq O_2_/Kg oil and mg MDA/g oil, respectively. At the beginning of the experiment, peroxide values are at levels ranging between 0.2 and 0.32 meq O_2_/Kg oil while TBARS values range between 0.15 and 0.24 mg MDA/g oil as it is shown at Fig. 2. After the six-week incubation time at 37 °C, it is possible to see variations in both indicators.

For supplementary data files related with the analysis of walnut quality: Mendeley Data, V1, doi: 10.17632/rsvsbvnvr4.1. Supplementary data were obtained from three independent replicates for each condition.

## 2 Experimental Design, Materials and Methods

### 2.1 Materials

Unshelled butterfly walnuts (Juglans regia L., Chandler variety) from trees cultivated on Requinoa, Libertador Bernardo O'Higgins Region (lat. 34° 16′ 43″ S, long. 70° 48′ 42″ W, altitude 416 m) were obtained for this study (2019 crop) and packaged (EPBN75 from Empack Company) with modified atmosphere (100% nitrogen). The final format of each bag was 350 g of walnuts, which were storage at 16°C and protected from the light until they were used to the coating experiments.

### 2.2 Edible coatings preparation

Edible coating was prepared following the method conducted by [1], with slight modifications. The thickeners in the formulation of edible coatings (carboximethylcellulose (CMC) or hydroxipropilmethylcellulose (HPMC)) were dispersed in distillate water (6.0 g/100 mL) under stirring conditions at 70 °C for 60 min. Then, the pH of the solution was adjusted to 9 using 0.1 N sodium hydroxide. Finally, glycerol (10 g/100 g solution) and vitamin E (tocopherol) at 0.5% (w/v) were added to the solution under stirring conditions as a plasticizer and antioxidant respectively. The coating forming solution was cooled at room temperature and was centrifuged (1186 g for 6 min) to obtain the final formulation.

### 2.3 Walnut's coating procedure and storage stability study

Walnuts were extracted from the packaging and separated in 9 groups of 350 g. Each group of walnut was immersed on each edible coating formulation, including distilled water for the control group. Walnuts were submerged for 30 s with slight movement to ensure complete coverage of their surface, draining for 3 min and air-dried at 16 °C for 24 h. The coated walnuts and control samples were then directly analysed or bagged and put at 37 °C (and 20%RH) in an incubator (Biochemical Incubator, SHP-350) for six weeks.

### 2.4 Peroxide Value (PV)

The peroxide value was determined by the standard AOCS Cd 8–53 methods and calculated in terms of meq of oxygen per kg of extracted oil. One gram of walnut lipid was mixed with 35 mL of 2:3 (v/v) chloroform/acetic acid solution. A saturated solution of potassium iodide was added and the solution was stirred in a dark room for 5 min, after which 75 mL of distilled water was added and mixed. 1 mL of starch solution (1 g/100 mL of distilled water) was added as indicator and the mixture was titrated with a normalized NaS_2_O_3_ solution at 0.01 N. The end point of the titration was determined by the disappearance of purple colour and the quantification of titration volume.

### 2.5 Thiobarbituric Acid Reactive Substances (TBARS)

This methodology is based in [2]. 2.5 mL of walnut oil and 2.5 mL of TBA reagent (46 mM in 99% glacial acetic acid) were mixed in a test tube and heated in a boiling water bath for 35 min. The reaction mixture was chilled, and the absorbance was measured at 532 nm using a Jasco UV−vis spectrophotometer. For quantification, standard solutions of malondialdehyde (MDA) in 7.5% trichloroacetic acid (TCA) were prepared from 1,1,3,3-tetraethoxypropane (TEP) and calibration curves were prepared at a concentration ranging from 0.6 to 10 µM.

## Ethics Statement

This work does not include any human subjects, animal experiments, or data from social media platforms.

## CRediT Author Statement

**Eduardo Caballero:** Conceptualization and design and coordination of activities of this work. Writing the final version of the article; **Daniela Bernal:** Data curation and analysis; **Jeniffer Wilckens:** Experimental methodology, writing the original draft.

## Declaration of Competing Interest

The authors declare that they have no known competing financial interests or personal relationships which have or could be perceived to have influenced the work reported in this article.
